# Expanding the pig metabolomics QTL catalog

**DOI:** 10.1186/s12711-026-01095-0

**Published:** 2026-10-21

**Authors:** Emil Ibragimov, Malene Kjelin Morsing, Martin Peter Rydal, Zexi Cai, Hans Stenlund, Jens Peter Nielsen, Merete Fredholm, Peter Karlskov-Mortensen

**Affiliations:** 1 Depatment of Veterinary and Animal Sciences, University of Copenhagen, København, Denmark; 2 Center for Quantitative Genetics and Genomics, Aarhus University, Aarhus, Denmark; 3 Department of Plant Physiology, Umeå University, Umeå, Sweden

## Abstract

**Background:**

Understanding the genetic regulation of metabolism is essential for improving animal health and productivity and for strengthening the use of pigs as translational models for human metabolic diseases. Metabolomics, when integrated with genomic data, provides a powerful framework for identifying genetic factors that influence intermediate molecular phenotypes closely linked to physiology. While large-scale metabolomics genome‑wide association studies (GWAS) have been extensively performed in humans, comparable studies in pigs remain limited. Among omics disciplines, metabolomics, the systematic study of metabolites central to physiology, represents the omics layer most closely linked to end-point phenotypes in mammals, such as health and production traits (e.g. growth rate). Metabolite levels are influenced by genetics, diet, and microbiome composition, with human studies showing high heritability and identifying numerous metabolite quantitative trait loci (mQTLs). While such findings have advanced our understanding of disease biology in humans, comparable studies in pigs remain limited.

**Results:**

We performed GWAS of the serum metabolome in 213 three‑way crossbred pigs [(Landrace × Yorkshire) × Duroc], analyzing 248 metabolites and 30,628 metabolite ratios. We identified 41 significant metabolite–variant associations, which were consolidated into 18 mQTLs based on linkage disequilibrium (LD). Several regions were associated with multiple, biochemically related metabolites, while others showed highly specific effects. Cross-species comparison with the human GWAS catalog revealed candidate genes within seven mQTLs. Through an integrative prioritization strategy, putative causal genes—predominantly enzymes and transporters—were identified for 14 of 18 mQTLs.

**Conclusions:**

Our results expand the catalog of genetic loci influencing serum metabolite variation in pigs and highlight both conserved and pig specific components of metabolic regulation. These findings provide a foundation for incorporating metabolomics into pig breeding programs and reinforce the value of pigs as a translational model for studying metabolite-associated traits and diseases in humans.

**Supplementary Information:**

The online version contains supplementary material available at https://doi.org/10.1186/s12711-026-01095-0.

## Background

Metabolites are small molecules that play a prominent role in animal physiology. Research dedicated to their investigation has become so crucial in life sciences that it has established its own field within the omics framework—metabolomics [1]. Within the hierarchy of omics technologies, metabolomics is among the closest layers to the end-point physiological phenotype [2]. In human studies, the impact of specific metabolites on disease and well-being have been demonstrated [3–5]. Thus, metabolomics provides a powerful link between molecular variation and organismal traits.

Metabolite levels are shaped by multiple factors, the most prominent being the composition of the microbiome [6, 7], diet [8, 9], and host genetics [10–16]. While the first two are beyond the scope of the present study, it is important to acknowledge that both nutrition and microbiome composition act as potential confounders in efforts to identify the genetic determinants of metabolite profiles. Recognizing these confounders is essential for correctly attributing variation in metabolites to genetic regulation.

Despite these confounding influences, human studies have shown that the heritability of metabolite levels remains high [12]. Numerous genetically influenced metabotypes (GIMs) and metabolite quantitative trait loci (mQTLs) have been identified. The accumulation of mQTL knowledge in humans has led to important discoveries, including causal effects of specific genetic variations on disease through a direct effect on intermediate metabolites [10, 14, 16], and the integration of metabolomics into predictive frameworks, such as multi-omic genetic scores for disease risk [15]. Furthermore, most causal genes underlying GIMs in humans were found to encode enzymes (~ 86%) or transporters (~ 7%) [10]. Together, these findings establish metabolomics as a genetically tractable and clinically informative layer of molecular data.

The pig (*Sus scrofa*) is not only a livestock species of high economic importance but also a valuable biomedical model due to its physiological similarity to humans [17]. Metabolites have been shown to influence pig health [18, 19] as well as important production traits, including feed efficiency [20–22], intramuscular fat content [23], and daily gain [24]. However, until recently, studies on the genetic determination of metabolite levels in pigs have been sporadic [25–27].

A breakthrough was achieved in 2025, when a large cohort of purebred pigs at slaughter age was analyzed for both genomic and metabolomic profiles [28]. This study demonstrated that some GIMs are conserved between humans and pigs, whereas others are unique to pigs. The former supports the use of pigs as a model for metabolite-driven human diseases, while the latter highlights the potential of pigs to provide novel insights into the genetic regulation of metabolism in mammals. Thus, pigs represent both a practical target for agricultural improvement and a translational model for human health research.

A complementary perspective was provided by Ponsuksili et al. [29], who investigated the genetic regulation of the fetal pig plasma metabolome. By focusing on metabolites measured during prenatal development, this study highlighted genetic effects acting at early developmental stages. The authors reported substantial genetic control of fetal plasma metabolites and identified loci with potential relevance for postnatal health and production traits. Together with studies in market‑weight pigs, these findings demonstrate that the genetic architecture of the porcine metabolome is stage‑dependent and can differ markedly between fetal and finishing stages.

In the present study, we extend the catalog of genetic determinants of metabolite levels in pigs. We analyzed integrated datasets of serum metabolite levels and genomic information from a three-way crossbred population [(Landrace × Yorkshire) × Duroc] sampled at an early post‑weaning stage (approximately two weeks after weaning). Genome-wide association studies (GWAS) were performed for both metabolite levels and metabolite ratios. The results were then characterized with respect to candidate causal genes and compared with findings from human studies. This approach provides novel insights into the genetic regulation of the metabolome in growing pigs, highlighting its significance for both animal production and biomedical research.

## Methods

### Animals

The study included 218 pigs (213 pigs after the filtering steps as described in the following subsections) originating from an experiment investigating post-weaning diarrhea previously described by Eriksen et al. and Ibragimov et al. [30, 31]. After genotype quality control (see Genotyping), 213 pigs were retained for the final analyses. The study was conducted between September and October 2019. Initially, 300 piglets originating from 30 sows (approximately 10 piglets per litter) were enrolled. All sows were inseminated using mixed semen from sires, resulting in a structured but genetically diverse population.

Antibiotic treatment of diarrhea during the two weeks prior to sampling was recorded and used as a covariate in the GWAS model. In total, 95 pigs received antibiotic treatment, of which 31 had recovered more than one week before sampling, 57 recovered within one week prior to sampling, and five pigs had not fully recovered on the day of sampling. The population consisted of 119 males and 94 females. Piglets were raised under conventional Danish commercial conditions in two cohorts (herds) housed on two different farms. Animals were three‑way crossbred, with Duroc as the paternal line and (Landrace × Yorkshire) as the maternal line. All pigs were born and raised in Denmark. Piglets were fed a standard commercial Danish weaner diet provided ad libitum, with free access to water at all times. Serum for metabolomic analysis was collected once per each pig at approximately five weeks of age during two sampling dates (one per each farm) using BD Vacutainer^®^ SST™ II tubes (3.5 mL, Silica). Samples were collected, kept on ice, centrifuged at 1400 × g, aliquoted, and stored at − 80 °C until further analysis.

### Genotyping

All animals were genotyped using the Porcine GeneSeek Genomic Profiler (GGP) 50 K single nucleotide polymorphism (SNP) panel (version 1) containing 50,697 SNPs. Quality control of animals was performed using PLINK (version 2.00) tool set [32]. This involved filtering out animals based on genotype missingness rate (< 0.05) using the “--mind 0.05” option and agreement between the farm-recorded sex and genotype-based sex; such animals were identified using the “--check-sex” command and subsequently removed using the “--remove” option. This step led to the removal of five animals, leaving 213 animals for analysis.

Prior to genome-wide association studies (GWAS), autosomal SNPs (*n* = 47,793) from the GGP 50 K panel were filtered based on minor allele frequency (MAF > 0.05; “--maf 0.05”), genotype missingness (< 0.05; “--geno 0.05”), and observed heterozygosity (< 0.95; identified from the “O(HET)” column in the output of “--hardy” and subsequently removed using the “--exclude” option). This filtering process retained 44,667 autosomal SNPs for downstream analysis. The heterozygosity filter was applied to mitigate biases arising from low sample counts within genotype groups, as Hardy–Weinberg equilibrium assumptions do not hold in three-way crossbred populations.

Genotypes were then imputed to whole-genome sequence level using a reference panel of the corresponding pig breeds, as described by Cai et al. [33]. Genotypes were phased using Eagle [34] and imputed in two steps with Minimac4 [35]. First, 50 K SNP genotypes were imputed to high‑density (HD) level using 474 (Landrace × Yorkshire) × Duroc pigs genotyped with the Affymetrix Axiom PigHD SNP array as an intermediate reference. Subsequently, HD genotypes were imputed to whole‑genome sequence level using 217 sequenced pigs from three DanBred commercial breeds (Duroc, Landrace, and Yorkshire). As a result, a total of 26,935,511 autosomal variants (imputed and genotyped) were obtained. Post‑imputation filtering retained variants with imputation accuracy (R² > 0.4), removing 4,255,944 low‑quality variants. The mean imputation accuracy for the retained variants was 0.947 (SD = 0.104). These high‑quality SNPs were further filtered using the same thresholds as for panel SNPs (MAF > 0.05, missingness < 0.05, and observed heterozygosity < 0.95), resulting in a final dataset of 14,968,568 variants for the second stage of GWAS (see “Genome‑wide association studies (GWAS)”).

### Metabolome screening

#### Sample preparation

Metabolite extraction of serum based on Aa et al. [36] was performed with a semi-automated approach using an Agilent Bravo automatic liquid handling platform (G5563AA, Agilent Technologies, CA, USA) with Agilent VWorks software for automation control. 720 µL of extraction buffer (90:10, v/v methanol: water) was dispensed into extraction plates (96 Agilent 203426-100 PP, 1 mL round-bottom). Internal standards LC-MS were included in the buffer (phenylalanine-13C9, caffeine-13C3, cholic acid-D4, caffeic acid-13C9, salicylic acid-D6). 80 µL of serum sample was added to each well, and mixing was performed using the Bravo system (1000 rpm, 10 cycles). Proteins were precipitated at -20 °C for 2 h, and the plates were centrifuged at 4 °C, 4000 rpm (3488 g) for 10 min in a tabletop swing-out rotor centrifuge (Mega Star 3.R, VWR Radnor, PA, USA). For LC-MS analysis, 200 µL of supernatant was transferred to LC plates (96 Agilent WP 5043–9314, 0.33 mL V-bottom) using the Bravo system. The samples were stored at -80 °C until analysis. Extraction blanks were prepared in the same way as the samples, using 80 µL water. Small aliquots of the remaining supernatants were pooled to generate quality control (QC) samples, which were aliquoted and injected at regular intervals to monitor data quality. QC samples were also analyzed by LC-MS/MS for metabolite identification. The samples were analyzed in batches according to a randomized run order.

#### LC-MS analysis

Before LC–MS analysis, the sample was re-suspended in 10 + 10 µL methanol and water. Samples were analyzed first in positive ion mode, followed by a second injection in negative ion mode. Chromatographic separation was performed on an Agilent 1290 Infinity UHPLC system (Agilent Technologies, Waldbronn, Germany). 2 µL of each sample was injected onto an Acquity UPLC HSS T3 C18 column (2.1 × 50 mm, 1.8 μm) with a VanGuard precolumn (2.1 × 5 mm, 1.8 μm; Waters Corporation, Milford, MA, USA) held at 40 °C. The gradient solvents were A: H_2_O with 0.1% formic acid, and B: acetonitrile/2-propanol (75:25, v/v) with 0.1% formic acid. The flow rate was 0.5 mL min^–1^. Elution was performed with a linear gradient of 0.1–10% B over 2 min, increased to 99% B over 5 min, held for 2 min, reduced to 0.1% B over 0.3 min with a simultaneous increase in flow rate to 0.8 mL min^–1^ for 0.5 min, held for 0.9 min, and then returned to 0.5 mL min^–1^ for 0.1 min before the next injection. Detection was carried out on an Agilent 6546 Q–TOF mass spectrometer equipped with a jet stream electrospray ion source operating in positive or negative ion mode. The same settings were used for both modes, except for the capillary voltage. A reference interface was connected for internal mass calibration using purine (4 µM, m/z 121.05 and 119.03632) and HP-0921 (1 µM, m/z 922.0098 and 966.000725) infused at 0.05 mL min^–1^. The gas temperature was 150 °C, drying gas flow 8 L min^–1^, and nebulizer pressure 35 psig. The sheath gas temperature was 350 °C with a flow rate of 11 L min^–1^. The capillary voltage was 4000 V in both modes, the nozzle voltage 300 V, the fragmentor voltage 120 V, the skimmer 65 V, and OCT 1 RF Vpp 750 V. Collision energy was set to 0 V. Data were acquired in centroid mode over m/z 70–1700 at 4 scans s^–1^ (1977 transients per spectrum).

#### Data preprocessing

LC-MS data were processed using a targeted, library-based workflow implemented in Agilent MassHunter Profinder version B.10.0.2 (Agilent Technologies Inc., Santa Clara, CA, USA). An in-house LC-MS library, constructed mainly from authentic standards analyzed on the same system under identical chromatographic and mass spectrometric conditions, was searched using the Batch Targeted Feature Extraction workflow with an m/z tolerance of 20 ppm and a retention time window of 0.1 min. The library contained compound-specific information, including accurate MS1 mass, retention time, and MS/MS fragmentation spectra. Candidate metabolite annotations obtained through this workflow were subsequently subjected to manual inspection and curation by experienced personnel, with each metabolite feature evaluated individually for consistency with the corresponding reference standard.

Confidence levels for metabolite annotation were assigned according to established community systems [37–39]. Five levels were defined: Level 1 (confirmed identification), based on direct comparison with an authentic reference standard analyzed under identical conditions, requiring agreement in accurate mass, retention time, and usually MS/MS spectra; Level 1* (isomeric mixture), applied when standards confirmed a mixture of structural isomers without chromatographic or MS/MS separation; Level 2 (putative annotation/structure), based on spectral library matches and/or diagnostic MS/MS evidence but without a standard; Level 3 (tentative annotation/compound class), based on accurate mass, isotope distribution, and database searches, enabling tentative structure or class assignment; Level 4 (molecular formula), features with accurate mass and molecular formula but insufficient evidence for structural assignment; and Level 5 (unique feature), based on accurate mass only, without a confirmed molecular formula.

A total of 248 metabolites were detected and successfully annotated using the targeted workflow, of which 231 were assigned annotation confidence Level 1, 16 Level 1*, and one Level 3, and those metabolites were used for the downstream analysis.

For GWAS, metabolite levels were processed as follows. First, the original metabolite matrix was log-transformed using NumPy [40]. Batch effects arising from the LC-MS plates were then corrected using the pyComBat function from the combat package [41].

Outlier detection was performed at the level of individual observations (i.e., per metabolite and per sample). Values deviating by more than three standard deviations from the metabolite‑specific mean were considered outliers and removed. No entire samples or metabolites were excluded based on missingness or outlier criteria. The resulting matrix was standardized using StandardScaler from Scikit-learn [42]. Alongside individual metabolite levels, we also computed and evaluated metabolite ratios. Metabolite ratios can capture biologically relevant relative changes between metabolites, such as product–substrate relationships, conjugation states, or shared regulation by enzymes or transporters. Ratios can also reduce the impact of shared technical and physiological variability, including dilution effects and uncontrolled factors such as time since last meal. All possible metabolite pairs were used to compute ratios, which were subsequently evaluated in a hypothesis‑free metabolite ratio genome‑wide association study (GWAS), because pathway annotation in pigs remains incomplete and restricting ratio selection to predefined biochemical relationships could bias discovery. For metabolite ratios, log-transformed ratios were calculated for all possible pairs of metabolites.

### Genome-wide association studies (GWAS)

GWAS was performed in two stages, both using additive linear mixed models implemented in GCTA (version 1.93.2) [43] with the leave-one-chromosome-out (LOCO; “--loco-mlma” option) approach, in which the genomic relationship matrix (GRM) is constructed excluding variants from the chromosome under test. In each GWAS, sex, cohort (herd), antibiotic treatment within the last two weeks, age, and body weight were included as fixed effects, and for metabolite ratios, LC–MS plate (batch effect) was additionally included. These covariates were incorporated in the GCTA model using the “--covar” (discrete variables) and “--qcovar” (continuous variables) options, such that metabolite and metabolite ratio levels were adjusted for the mean and fixed effects within the linear mixed model, and the resulting residual variation was used to test genetic variant associations according to Eq. (1):

1 $$\:\mathbf{y}=b\mathbf{x}+{\mathbf{g}}^{-}+\mathbf{e}$$

where **y** is the vector containing the adjusted metabolite/metabolite ratio levels (i.e., residuals after accounting for fixed effects), *b* is the additive effect of the candidate variant, **x** is the candidate variant genotype indicator variable codded additively (0, 1, or 2), **g**^−^ is the accumulated effect of all variants (i.e., GRM) except the chromosome, on which candidate variant is located and e is the residual, **e** is the vector of residuals.

The genome-wide significance threshold was defined separately for individual metabolites and metabolite ratios, using a significance level (α = 0.05) corrected for the number of independent variants and independent traits. The number of independent variants was estimated as the number of variants in linkage disequilibrium (*r*²) below 0.75 [44], determined using the ‘--indep-pairwise 50 5 0.75’ procedure in PLINK. The number of independent tests for metabolites and ratios was estimated following the method described in the study on the genomic atlas of the plasma metabolome [10], as previously applied by Wang et al. [45]. In short, we estimated the effective number of independent phenotypes by performing an eigen decomposition of the phenotype covariance matrix and defining it as the number of traits that would explain the same total variance if all traits were uncorrelated. Eigenvalues were calculated using the eigvalsh function in NumPy [40]. The inferred number of effective tests was 21.29 for metabolites. For metabolite ratios, the number of effective tests was assumed to equal the square of the number of effective metabolite tests, resulting in 453.26 tests. As result, we estimated p-value threshold as 9.14 × 10⁻⁸ for metabolites and as 4.29 × 10⁻⁹ for ratios.

The first stage was performed for each metabolite and each metabolite ratio but only including the ~ 45 K autosomal SNPs, genotyped using the SNP-chip. The second stage analyzed WGS level imputed genotype data, but only for chromosomes identified as harboring significant variants for specific metabolites or ratios in stage 1, and then only for those specific metabolites or ratios, while keeping the same significance thresholds as in the stage 1. This approach significantly reduced the required computer time. For each chromosome–trait pair, the minimal p-value was recorded, and pairs with values below the genome-wide significance threshold were included in the second stage analyses. For metabolite ratios, significance was additionally evaluated using the p-gain criterion, defined as the ratio of the minimal GWAS p-value of the lead variant for the numerator and denominator traits to the GWAS p-value for the corresponding metabolite ratio, with a threshold of ≥ B/(2*α), where α is the level of significance (0.05) and B is the number of independent tests for metabolite ratios (453.26) [46]. To identify multiple independent associations within the same chromosome, we performed a conditional analysis by running another round of GWAS including the lead variant genotype as a covariate.

Association regions were defined based on the positions of the variants in high linkage disequilibrium (LD, *r*² > 0.8) with the lead variant, extending to the furthest such variants on either side. When association regions for different traits overlapped, they were merged into mQTLs for further analyses. The borders of each mQTL region were defined by the leftmost and rightmost boundaries of the constituent trait-specific association regions and were further extended by 500 kb on each side. The identified mQTLs were used to identify candidate causative variants and make comparisons with the human GWAS catalog.

#### Linkage disequilibrium (LD) and correlation analysis

LD (*r*^2^) between lead genetic variants was evaluated using the “--r2” function in PLINK. For correlation analyses, metabolite and metabolite ratio levels were adjusted for the same fixed effects as those used in the GWAS using the LinearRegression function from Scikit-learn (v. 1.9.0) [42]. Correlations between traits were then estimated as the absolute values of Pearson correlation coefficients. Pairwise LD matrices were visualized as heatmaps using the heatmap function in Seaborn [47], while trait correlation matrices were visualized with the clustermap function from Seaborn.

#### Variant effect prediction

Genetic variants that reached the significance threshold and were located within mQTL regions were further examined for their potential impact on genes. Only single nucleotide variants (SNVs) were considered, while indels were excluded. Variant effects were predicted using SnpEff [48], and only those classified with at least a moderate impact on the corresponding gene were retained. Variant impact categories (‘LOW’, ‘MODERATE’, ‘HIGH’) were assigned using SnpEff, where ‘MODERATE’ includes non-disruptive variants such as missense mutations that may alter protein effectiveness, while ‘HIGH’ includes variants predicted to cause protein truncation or loss of function. For reporting, we have considered only variants which have p-value not more than 10 times bigger than the minimal p-value among all variants passed inclusion criteria per mQTL (to have not less than moderate impact) in the main table, while additional candidate genes are provided in the supplementary materials.

#### Human GWAS catalog

To identify overlaps with human studies, we used the defined mQTL regions and searched for corresponding phenotypes in the human GWAS Catalog (version 1.0.2) [49]. This was done by semi-manual curation of the catalog entries (columns MAPPED_TRAIT and DISEASE/TRAIT), retaining only terms that showed similarity to the representative traits for mQTL. After mapping traits, the associated genes were extracted from the GWAS Catalog, and their orthologous counterparts in *Sus scrofa* were identified. If an orthologous gene was located within the corresponding mQTL region, the overlap is reported.

#### Candidate gene ranking based on functional relevance

To prioritize functional candidate genes within mQTL regions, we focused on enzymes and transporters in the *Sus scrofa* genome. First, all genes annotated as enzymes or transporters were identified using the Gene Ontology (GO) database [50, 51]. To achieve this, all child terms of catalytic activity (GO:0003824) and transporter activity (GO:0005215) were extracted, and genes associated with these terms were retrieved using the GO web browser. Genes located within mQTL regions were then annotated based on Ensembl (release 115) [52] and cross-referenced with the results of the GO-based gene list. The extracted list of transporters and enzymes was manually curated to identify plausible associations between genes and the representative traits affected by each mQTL. In cases of ambiguity where more than one gene appeared relevant, prioritization was guided by predicted variant effects and evidence from the human GWAS Catalog.

## Results

### The genome-wide association studies (GWAS) reveal genetic factors that influence metabolite levels

The metabolomics dataset comprised 248 annotated serum metabolites spanning a broad range of biochemical classes [Additional file 1, Table S1]. These included 71 amino acid–related metabolites (amino acids and derivatives), 62 lipids (predominantly glycerophospholipids and sphingolipids), 48 fatty acids and related organic acids, 22 nucleic acid–related metabolites (nucleobases, nucleosides, and nucleotides), 16 carnitines and acylcarnitines, 12 carbohydrates and sugar acids, 10 bile acids, and 7 benzenoid compounds. This composition provides broad coverage of central metabolic pathways relevant to energy metabolism, amino acid turnover, lipid metabolism, and host–microbiome interactions.

Our GWAS of 248 metabolites and 30,628 metabolite ratios using the GGP 50 K SNP panel identified metabolite-variant associations for 15 metabolites and 24 metabolite ratios at genome-wide significance (p-value < 9.14 × 10⁻⁸ for metabolites; p-value < 4.29 × 10⁻⁹ for ratios).

To refine these signals, we conducted a second GWAS using sequence-level imputed genotypes for the chromosomes harboring metabolite-associated variant(s), applying the same significance thresholds. Due to long-range LD and the relatively low genetic heterogeneity of pig populations, imputation often leads to clusters of variants with identical p-values rather than discrete lead variants. Instead of pruning such regions, we report “lead variant ranges” to better reflect the spread of top associations within genomic intervals. Finally, to detect independent signals within the same chromosome, we ran a second round of GWAS while including the identified lead variants as fixed effects. This second round of GWAS revealed one more independent association for metabolite level on the same chromosome.

**Fig. 1:**
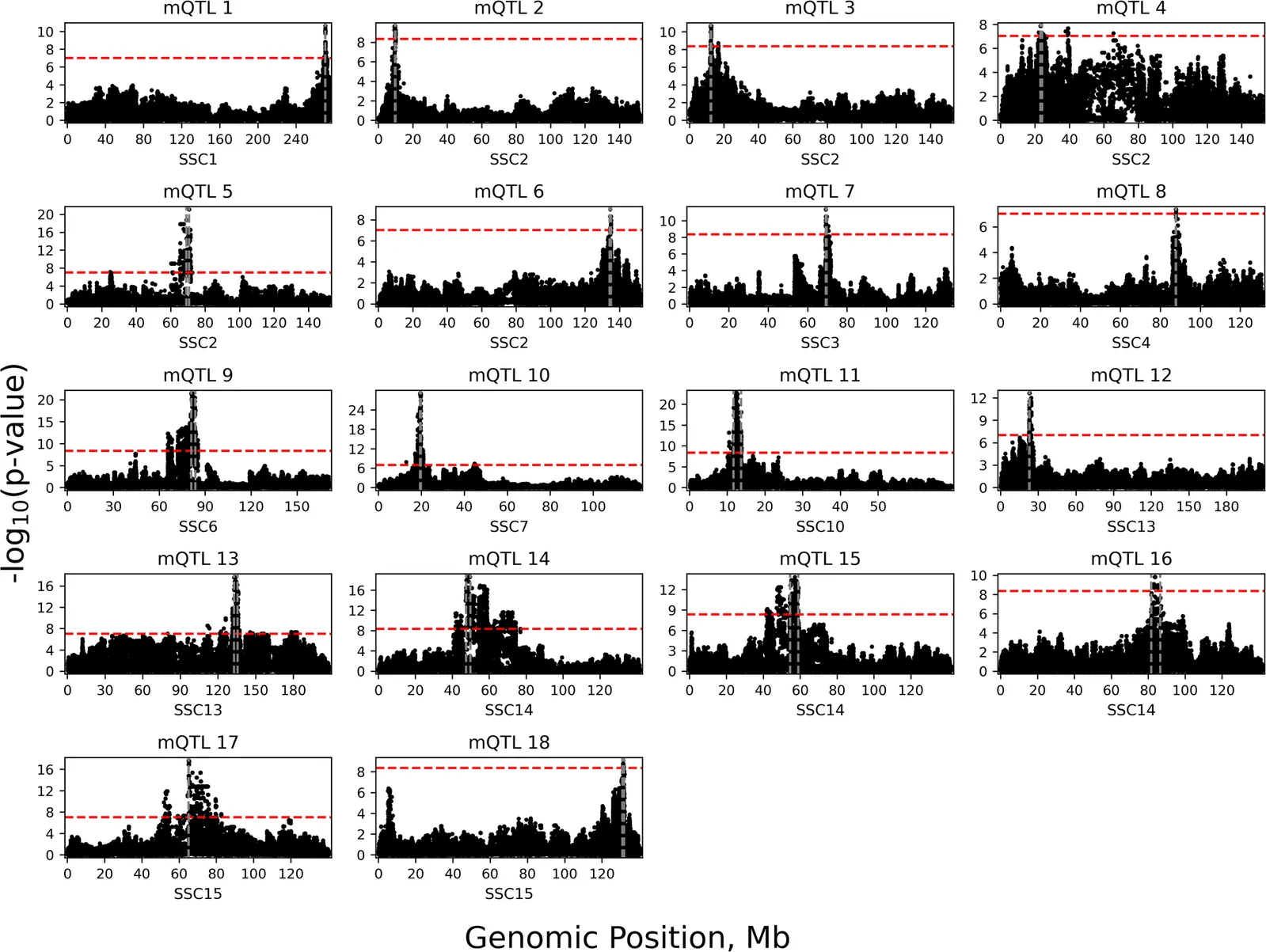
Genome‑wide association results for mQTL regions. Manhattan plots are shown for the chromosome harboring each merged mQTL region. When a merged mQTL was associated with multiple metabolic traits, the trait exhibiting the strongest association signal (lowest GWAS p‑value) was selected as the representative trait for visualization. The horizontal red dashed line highlights the genome-wide significance level; grey vertical dashed line indicates mQTL borders

Thus, in total, we identified 41 significant metabolite–variant associations for metabolites and metabolite ratios. The boundaries of each trait-specific association region were defined as the leftmost and rightmost variant in high LD with the lead variant (*r*² > 0.8) [Additional file 1, Table S2]. Several trait-specific association regions either shared the same lead variant or showed overlapping genomic regions. To provide a clearer overview, such regions were merged into one mQTL [Additional file 1, Table S3], what resulted in 6 complex (explaining more than one metabolic trait) mQTLs and 12 independent mQTLs for single metabolites or ratios. For each of the 18 mQTL regions, a Manhattan plot was generated; in regions associated with multiple traits, the trait showing the lowest GWAS p‑value was selected as the representative trait (Fig. 1).

Associations grouped into mQTL10 and mQTL 11 shared the same lead variant, while the rest four mQTLs contained from two to three lead variants. Thus, we further examined the LD structure within mQTLs containing more than one trait-specific association [Additional file 2, Figure S1], and all lead variants across a given mQTL were in high LD (*r*² > 0.67), consistent with a single underlying association signal.

**Fig. 2:**
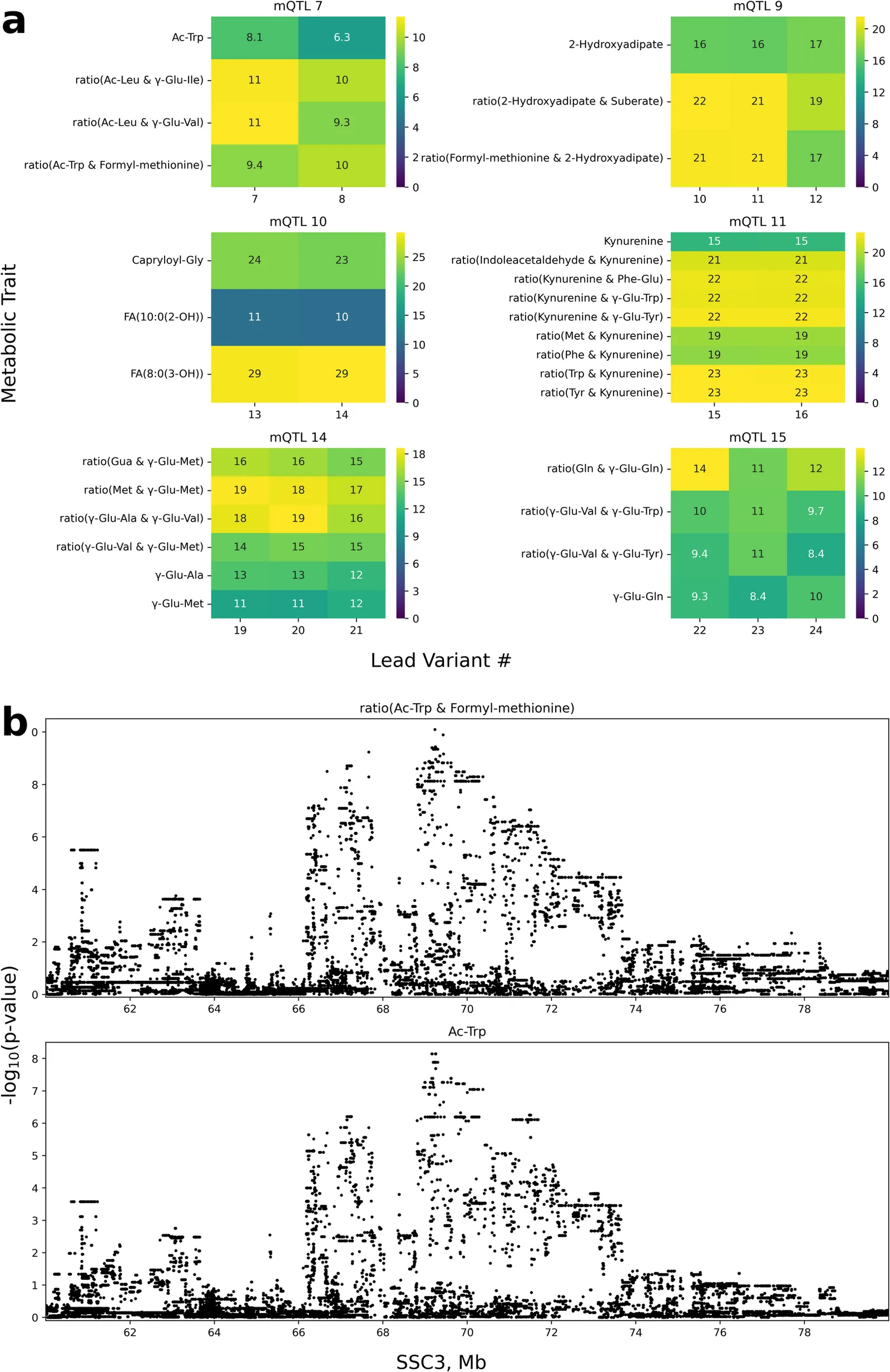
Cross‑trait association patterns within mQTL regions. (**a**) Heatmap showing the strength of association (− log10 (p-value)) for all metabolic traits mapped to the same mQTL region across all trait‑specific lead variants within that region. Lead variants are labeled by variant identifier (see [Additional file 1, Table S2] for details). (**b**) Regional GWAS plots for two metabolic traits associated with mQTL 7, illustrating asymmetric association strength between traits while supporting a shared underlying genetic signal

To assess whether metabolic traits mapped to the same pleiotropic mQTL region reflect a shared underlying association signal despite having different lead variants, we systematically evaluated the effect of each trait‑specific lead variant across all traits associated with that region (Fig. 2a). In most mQTL regions, lead variants identified for individual traits showed significant associations with the other traits within the same region, with differences in association strength being relatively modest.

However, we also observed cases of asymmetric association strength. For example, in mQTL 7, the lead variant identified for the ratio between acetyl‑tryptophan and formyl‑methionine did not reach genome‑wide significance for acetyl‑tryptophan alone, whereas the lead variant for acetyl‑tryptophan showed a genome‑wide significant association with the ratio. To further illustrate this pattern, we visualized the full GWAS results for both traits in this region (Fig. 2b), demonstrating that these associations likely reflect a shared genetic signal with unequal effect sizes rather than distinct loci.

The identified mQTLs were associated with up to nine metabolic phenotypes. To explore potential pleiotropic effects, we first examined phenotypic correlations among metabolite and metabolite ratio levels (after adjustment for the fixed effects) controlled by the same mQTL. These correlations ranged from weak (|*r*| ~ 0.2) to extremely strong (|*r*| > 0.95) [Additional file 2, Figure S2], indicating substantial heterogeneity in the degree of phenotypic similarity among traits associated with the same mQTL region.

### Imputed variants representing candidate causative mutations

Imputed data facilitated identification of potential causal mutations in our data set. To assess their functional impact, we predicted effects of variants, which passed genome-wide level of significance, as described in Methods. Variants with “MODERATE” to “HIGH” impact at the gene or protein levels were retained. This resulted in the identification of 88 genome-wide significant SNPs, but not necessarily lead-variants, with potential effect on a gene [Additional file 1, Table S4]. In total, we identified 46 genes harboring variants with predicted functional impact across 11 mQTL regions, whereas seven mQTL regions did not contain variants meeting these criteria. To shorten the list, we have prioritized the genes which contained impactful variant with lowest p-value as described in the Methods section (Table 1).

**Table 1:** Top genes harboring candidate causative genetic variant

mQTL	mQTL region	Representative trait(s)	Gene name	*p*-value
1	1:270,519,470 − 270,547,642	Cit	*HMCN2*	1.44e-09
2	2:9,614,591-9,863,471	ratio(LPC(20:3/0:0) & LPC(22:3/0:0))	*FADS1*	1.2e-09
			*ENSSSCG00000013078*	1.87e-09
3	2:12,230,448 − 12,557,385	ratio(2-Me-Butyryl-Gly & Eicosanedioate)	*GLYATL2*	2.04e-11
			*FAM111B*	9.5e-11
9	6:80,893,989 − 83,607,466	2-Hydroxyadipate	*HMGCL*	3.5e-22
			*KDM1A*	3.5e-22
			*ENSSSCG00000025440*	5.22e-22
			*RHCE*	3.14e-21
10	7:19,292,058 − 19,766,720	FA(8:0(3-OH)) & Capryloyl-Gly & FA(10:0(2-OH))	*ACOT13*	9.1e-30
			*TDP2*	9.1e-30
11	10:11,647,390 − 13,688,316	Kynurenine	*NVL*	4.02e-21
12	13:22,857,478 − 23,059,659	Quinate	*PLCD1*	8.67e-10
			*VILL*	8.67e-10
			*ACAA1*	2.56e-09
			*DLEC1*	2.56e-09
13	13:132,734,666 − 135,533,132	2,4-DHBA	*ENSSSCG00000062521*	1.29e-17
14	14:47,424,622 − 49,776,312	γ-Glu-Met & γ-Glu-Ala	*ENSSSCG00000035443*	1.85e-18
15	14:54,474,260 − 58,862,819	γ-Glu-Gln & γ-Glu-Val	*TARBP1*	3.99e-14
17	15:65,013,358 − 65,038,063	dU	*ENSSSCG00000015876*	3.08e-18

Columns: mQTL number; mQTL region (Chromosome and borders); representative traits; gene name with candidate causative variant; minimal p-value among detected variants

### Seven mQTLs are in agreement with results from human studies

To identify overlaps with human phenotypes, we first matched human phenotype annotations to our mQTLs (a complete list is provided in [Additional file 1, Table S5]). In the final results, we retained only overlaps where genes identified in human studies were located within the corresponding mQTL region. Using this approach, we identified candidate genes for seven mQTLs, encompassing a total of 30 unique genes (Table 2 and [Additional file 1, Table S6]).

**Table 2:** Genes verified by human studies

mQTL	Representative trait(s)	Gene name	References
1	Cit	*ASS1*	[10, 53, 54]
		*HMCN2*	[14, 16]
2	ratio(LPC(20:3/0:0) & LPC(22:3/0:0))	*BEST1*	[55–57]
		*DAGLA*	[56, 57]
		*FADS1*	[53, 55–62]
		*FADS2*	[53, 55–64]
		*FADS3*	[56, 57]
		*FEN1*	[53, 56, 57]
		*MYRF (ENSSSCG00000013078)*	[53, 56, 57]
		*RAB3IL1*	[55–57, 59]
		*SYT7*	[56, 57]
		*TMEM258*	[53, 56, 57, 60, 61, 63]
6	Trp-Bet	*IRF1*	[54]
		*P4HA2*	[16, 65]
		*RAD50*	[54]
		*SLC22A4*	[10, 65]
		*SLC22A5*	[10, 14, 54, 65–68]
7	Ac-Leu & Ac-Trp	*ACTG2*	[54]
		*ALMS1*	[10, 14, 16, 54, 65, 67, 69]
		*CCT7*	[54]
		*DUSP11*	[16]
		*NAT8/NAT8B*	[10, 14, 16, 54, 62, 65–67, 70]
		*NOTO*	[54]
		*RAB11FIP5*	[54]
		*STAMBP*	[54, 69]
11	Kynurenine	*KMO*	[10, 14, 53, 67–69
13	2,4-DHBA	*PCYT1A*	[10, 16, 54, 67]
		*SLC51A*	[10, 16, 54, 67]
14	γ-Glu-Met & γ-Glu-Ala	*GGT1 (ENSSSCG00000010056)*	[14, 67, 68]

### Most mQTLs harbor genes encoding relevant transporters or enzymes

Finally, we tried to prioritize the most likely causal gene for each mQTL among enzyme- and transporter-encoding genes located within the respective regions. For this purpose, we manually assessed the functional relevance of all such genes to the corresponding metabolites. When this relevance was further supported by evidence from homologous human regions and variant effect predictions indicating potential causative variants, we prioritized these gene–metabolite pairs. Using this approach, we identified candidate genes for 14 out of 18 mQTLs (Table 3).

**Table 3:** Prioritized enzyme/transporter encoding genes per mQTL

mQTL	mQTL region	Representative trait(s)	Gene	Human GWAS Catalog^*^	SnpEff^**^	Enzyme/Transporter^***^
1	1:270519470–270,547,642	Cit	*ASS1*	+	-	E
2	2:9,614,591-9,863,471	ratio(LPC(20:3(/0:0),LPC(22:3/0:0))	*FADS1*	+	+	E
3	2:12,230,448 − 12,557,385	ratio(2-Me-Butyryl-Gly & Eicosanedioate)	*GLYATL2*	-	+	E
			*GLYAT*	-	-	E
6	2:134,213,624 − 134,985,008	Trp-Bet	*SLC22A4/SLC22A5*	+	-	T
7	3:69,156,521 − 69,615,490	Ac-Leu & Ac-Trp	*NAT8 (ENSSSCG00000021579)*	+	-	E
10	7:19,292,058 − 19,766,720	FA(8:0(3-OH)) & Capryloyl-Gly & FA(10:0(2-OH))	*ACOT13*	-	+	E
11	10:11,647,390 − 13,688,316	Kynurenine	*KMO*	+	+	E
12	13:22,857,478 − 23,059,659	Quinate	*SLC22A13/SLC22A14*	-	-	T
13	13:132,734,666 − 135,533,132	2,4-DHBA	*SLC51A*	+	-	T
14	14:47,424,622 − 49,776,312	γ-Glu-Met & γ-Glu-Ala	*GGT1 (ENSSSCG00000010056)*	+	-	E
15	14:54,474,260 − 58,862,819	γ-Glu-Gln & γ-Glu-Val	*MTR*	-	-	E
16	14:81,629,959 − 86,537,789	ratio(Met, Trp)	*MAT1A*	-	-	E
17	15:65,013,358 − 65,038,063	dU	*UPP2*	-	+	E
18	15:130,926,414 − 131,797,491	ratio(CAR(16:0) & CAR(18:2))	*SLC16A14*	-	-	T

^*^Column listing whether the metabolite–gene pair is supported by evidence from the human GWAS Catalog (‘+’ = supported); ^**^ Column listing whether the metabolite–gene pair is supported by an impactful variant detected using SnpEff (‘+’ = supported); ^***^ E-enzyme, T - transporter

## Discussion

In this study, we implemented an integrated metabolomics–genomics pipeline to dissect the genetic architecture of serum metabolite variation in crossbred pigs. As a result, we identified 41 metabolite–variant associations, which we consolidated into 18 distinct mQTLs.

Interestingly, several metabolites and metabolite ratios associated with the same mQTL region exhibited only weak phenotypic correlations. While a shared association signal may reflect either pleiotropic effects of a single causal variant or multiple linked causal variants within the same genomic region, the biochemical characteristics of the associated traits provide additional context. For example, all metabolites and metabolite ratio traits associated with mQTL 7 contained an acetyl group, whereas all traits associated with mQTL 15 contained a glutamyl group. Although these observations do not establish the underlying mechanism, they suggest that the shared genetic signals may, at least in part, reflect effects on related biochemical processes despite limited phenotypic covariance among the corresponding traits.

By using imputed genotypes, we were able to identify potential causal variants. Initially, we identified 46 candidate genes carrying variants with predicted functional effects; however, due to extensive long-range LD, there is a risk of false positives. To minimize this, we combined information from the human GWAS catalog with manual curation of gene–metabolite pairs and ultimately prioritized five out of 46 genes with clear functional relevance.

However, we cannot rely on predicted variant effects to infer causality. Associations may also arise from regulatory mechanisms that alter gene expression rather than from changes in coding regions. Notably, at least nine promising candidate genes in our study did not contain coding variants with potential impact, suggesting that causal mutations may lie in regulatory regions, as previously discussed in human and pig metabolomics studies [10, 28, 66]. This underscores the importance of integrating regulatory genomics, such as eQTL and chromatin accessibility data, into future mQTL studies.

Seven of the mQTLs identified in pigs were also supported by human studies. For some of these overlapping loci, the genes encoding enzymes or transporters harbored functionally relevant variants (e.g., fatty acid desaturase 1 for the LPC(20:3/0:0) to LPC(22:3/0:0) ratio, and kynurenine 3-monooxygenase for Kynurenine). Such shared genetic regulation of metabolite levels provides strong support for the use of pigs as model organisms to study metabolite-driven diseases. Notably, several identified candidate genes are already known to be associated with human disorders. For example: argininosuccinate synthase 1 (*ASS1*) is linked to citrullinemia [71]; kynurenine 3-monooxygenase (*KMO)* has been implicated in Huntington’s and Alzheimer’s disease [72], and gamma-glutamyltransferase 1 *(GGT1)* is associated with glutathionuria [73], and N-acetyltransferase 8 *(NAT8)* is associated with chronic kidney disease (CKD) [74].

While many of our results are consistent with previous studies in humans, we also refined gene prioritization within specific mQTL regions. For example, mQTL 10, which is associated with 3‑hydroxyoctanoic acid, capryloyl‑glycine, and 2‑hydroxycapric acid, harbors variants with predicted functional impact on acyl‑CoA thioesterase 13 *(ACOT13)*. Although *ACOT13* has not been directly linked to metabolite traits in the human GWAS Catalog, acyl‑CoA thioesterases are known to regulate mitochondrial medium‑chain acyl‑CoA pools by hydrolyzing acyl‑CoAs to free fatty acids and CoA, thereby modulating β‑oxidation flux and preventing acyl‑CoA accumulation [75]. Perturbation of *ACOT13* activity is therefore expected to influence multiple downstream acyl‑CoA–derived metabolites, including hydroxylated medium‑chain fatty acids and acylglycines. This mechanistic framework is consistent with the observed pleiotropic association pattern and supports *ACOT13* as a strong functional candidate gene for this mQTL.

A second example is mQTL 17, which affects circulating deoxyuridine levels and contains variants with predicted impact on uridine phosphorylase 2 (*UPP2*). The link between UPP2 and deoxyuridine is direct and well supported biochemically: although UPP2 is classically described as catalyzing the phosphorolytic cleavage of uridine to uracil and ribose‑1‑phosphate, the enzyme exhibits broad substrate specificity that includes deoxyuridine [76]. Genetic variation affecting UPP2 activity is therefore expected to modulate deoxyuridine levels. Notably, we did not observe an association between this mQTL and uridine, despite uridine being measured in our dataset, suggesting differential regulation or substrate‑specific sensitivity within pyrimidine nucleoside metabolism.

Although not the first study of its kind [28], this is the first to investigate early-age crossbred pigs using serum metabolite profiling. Direct comparison with the study by Bovo and co-workers is limited, as the overlap in measured metabolites was small (32 of 248 metabolites). Nevertheless, two metabolites, Citrulline (Cit) and Kynurenine, were consistently associated with the same mQTL regions across both studies. These replicated associations indicate robust and reproducible genetic regulation of these metabolites, highlighting them as promising targets for further functional and comparative investigation.

Importantly, the novel mQTLs identified here are of equal interest to those overlapping with human studies, as they provide new insights into pathways of metabolite regulation. For seven novel mQTLs, we were able to propose strong candidate genes, whereas four remained uncharacterized. Several factors may explain the lack of gene prioritization in these regions. First, the homogeneity in genomic architecture of production animals and extensive haplotype structure may have influenced the precision of mQTL boundary definition. Second, causative genes may not conform to prior expectations; human studies have shown that ~ 6% of causal mQTL genes encode neither enzymes nor transporters [10]. In pigs, such “non-canonical” candidate genes may be overrepresented due to haplotype-mediated effects, where multiple genes with modest individual effects are inherited together in long LD blocks, and their combined impact can approximate that of an altered enzyme or transporter.

Another important aspect of our study is the extensive use of metabolite ratios as derived metabolic traits. Ratio‑based analyses have been widely applied in human metabolomics GWAS and have repeatedly demonstrated increased power to detect genetic associations compared with single‑metabolite analyses alone. Biologically, metabolite ratios can approximate enzymatic or transport steps, reflect conjugation or modification processes, or capture shared regulation of metabolites within the same pathway [10, 46, 66]. At the same time, ratio‑based analyses can introduce redundancy due to correlated metabolites; in this study, this was mitigated by applying a multiple‑testing‑corrected p‑gain threshold, as recommended in previous studies [46], and by merging overlapping association regions into fewer, more interpretable mQTL regions.

## Conclusions

This study identified 41 metabolite-variant associations in crossbred pigs, consolidated into 18 mQTLs. Seven of these overlapped with findings from human studies, indicating similarities between pigs and humans in metabolite-linked traits and diseases. The remaining loci represent novel mQTLs that expand our understanding of metabolite regulation; for seven of these, we were able to propose relevant candidate genes through integration of variant effect predictions and manual curation. Future studies should incorporate transcriptomics and whole-genome sequencing to improve the annotation of causative mutations and refine gene prioritization. Beyond livestock genetics, our results also provide a foundation for translational research into human health and nutrition.

## Supplementary Information

Below is the link to the electronic supplementary material.

Supplementary Material 1: Table S1. Annotation and classification of detected metabolites. Table S2. Summary of identified metabolite-specific association regions. Table S3. List of identified mQTL regions. Table S4. Variants significantly associated with gene expression or function. Table S5. Correspondence between mQTL regions and human GWAS traits (“GWAS Catalog entry type” specifies in which column of Human GWAS catalog database the given trait appears). Table S6. mQTL genes showing shared effects on related metabolites and human GWAS traits.

Supplementary Material 2: Figure S1. Linkage disequilibrium between lead variants in the same mQTL. Figure S2. Correlation between traits within same mQTL.

Supplementary Material 3

Supplementary Material 4

## Data Availability

All datasets generated or analyzed during this study are available from the corresponding author upon reasonable request.
