# Supplementary material for: Expanding the pig metabolomics QTL catalog
Source: Genet Sel Evol. 2026 Oct 21;58:75. doi: 10.1186/s12711-026-01095-0 (PMC13685178; doi:10.1186/s12711-026-01095-0)
Supplement: Supplementary file 1 [file 12711_2026_1095_MOESM2_ESM.docx]

Additional file 2

Expanding the Pig Metabolomics QTL Catalog

Emil Ibragimov^1^, Malene Kjelin Morsing^1^, Martin Peter Rydal^1^, Zexi Cai^2^, Hans Stenlund^3^, Jens Peter Nielsen^1^, Merete Fredholm^1^, Peter Karlskov-Mortensen^1^

^1^Depatment of Veterinary and Animal Sciences, University of Copenhagen, Denmark

^2^Center for Quantitative Genetics and Genomics, Aarhus University, Denmark

^3^Department of Plant Physiology, Umeå University

**Figure S1 - Linkage disequilibrium between lead variants in the same super-QTL.** Tick number indicate lead variant number as it appears in Table S2.

**Figure S2 – Correlation between traits within same super-QTL.** Tick numbers indicate metabolite number (see Table S1).
